# Canine olfactory detection of florfenicol residues in goat milk: a pilot study

**DOI:** 10.3389/fvets.2025.1579933

**Published:** 2025-07-21

**Authors:** Maaike O. Clapham, Dierdra McElroy, Melissa A. Mercer, Philip H. Kass, Lisa A. Tell

**Affiliations:** ^1^Department of Veterinary Medicine and Epidemiology, School of Veterinary Medicine, University of California, Davis, Davis, CA, United States; ^2^Laika Diagnostics, California Canine, Lathrop, CA, United States; ^3^Department of Population Health and Reproduction, School of Veterinary Medicine, University of California, Davis, Davis, CA, United States

**Keywords:** olfaction, drug residue detection, milk, goat, canine, florfenicol

## Abstract

**Introduction:**

Canine olfaction has been used to detect drug residues across a variety of matrices as part of law enforcement efforts. As such, canine olfactory sample screening should hold promise as a potential tool for detecting drug residues in food products to support human food safety in resource limited settings or where sensitive analytical methods are not available for various matrices. The objective of this pilot study was to evaluate the ability of companion dogs undergoing low-frequency olfactory detection training to detect florfenicol and its metabolite, florfenicol amine (FA), in incurred residue goat milk samples.

**Methods:**

Companion dogs (*n* = 8) of various breeds with prior odor detection experience were enrolled in a canine odor detection study for 9 weeks to detect florfenicol/FA that entailed once weekly testing sessions. Double-blinded testing was performed in two phases. Study phase 1 consisted of 11 florfenicol/FA-contaminated goat milk samples (combined [florfenicol + FA] concentrations ranging from 17.44–1443.30 ppb) with 2 distractors, items that might distract the dog while working, per run presented to *n* = 8 dogs. For study phase 2, the highest performing dogs (*n* = 3) from study phase 1 were tested with low concentration (<20 ppb) samples (*n* = 11) that were identified as being positive using a rapid residue detection test. Performance metrics, including accuracy, sensitivity, and specificity, were assessed across sample drug concentration categories.

**Results:**

For study phase 1, mean detection accuracy, sensitivity, and specificity were 0.80 [95% confidence interval (CI) (0.74–0.86), 0.70 (95% CI 0.65–0.76), and 0.86 (95% CI 0.82–0.88)], respectively. Sensitivity increased with higher drug concentrations, ranging from 0.38 at 17.96 ppb to 0.96 at 1443.30 ppb. Study phase 2 accuracy, sensitivity, and specificity were 0.88 (95% CI 0.85–0.91), 0.82 (95% CI 0.73–0.88), and 0.91 (95% CI 0.86–0.94), respectively. False positives were most often associated with blank goat milk.

**Discussion:**

Companion dogs undergoing low-frequency olfactory odor detection training were able to detect florfenicol/FA residues in goat milk with high specificity, particularly at high concentrations. However, sensitivity at low concentrations was limited. While canine olfactory detection does not appear to be suitable as a confirmatory method for companion dogs with low training commitments, this pilot study demonstrates its potential as an initial screening tool, particularly in resource-limited settings. Future research is needed to refine training protocols and assess performance under field conditions.

## 1 Introduction

Drug residue testing in matrices from food-producing animals is crucial for ensuring human food safety, yet it is often expensive and requires specialized training, laboratory equipment, and validated assay methods. These limitations can pose significant challenges for species or matrices where a validated assay does not exist, and for resource limited settings where access to analytical chemistry equipment and reagents may be limited, and where a lack of centralized testing authority might preclude adequate drug residue testing of food products even if established maximum residue limits exist (1, 2). Therefore, easily accessible and inexpensive methodologies for screening animal derived food products for drug residues could be useful in mitigating adverse human health consequences secondary to drug residue exposure. While most new methodologies for drug residue detection (such as immunoassays, biosensors, electrophoresis and molecular-based methods) have focused on increasing analytical sensitivity and developing multi-drug residue methods, there has been limited focus on developing inexpensive screening methods that do not require extensive equipment and specialty trained personnel (3). Goat milk is a nutrient dense and widely consumed food product in many parts of the world (4, 5). While producers generally have a high degree of awareness of the possibility of antimicrobial drug residues in goat milk products, antimicrobial drug residues are still highly prevalent in goat milk in resource limited regions with less intensive milk residue testing capabilities (6, 7). When contaminated with drug residues, milk could pose serious public health risks, especially in remote communities lacking the infrastructure and financial means to implement conventional testing methodology (8). To address this gap, unconventional approaches to drug residue detection in food products, such as the use of olfactory residue detection dogs, are gaining interest. While dogs have been successfully used in police, military, and medical detection tasks for decades, the use of olfactory detection dogs in the food safety field is a relatively new development (9, 10). Nonetheless, the olfactory capabilities and trainability of companion dogs make them a promising, low-cost candidate for preliminary detection of antibiotic residues in food products, like milk.

Working dogs are widely used for olfactory detection of a variety of chemical and volatile odors for civilian, military, medical, and forensic applications, with published detection thresholds in the range of parts per billion (ppb) to parts per trillion (ppt) (11–16). For rapid detection of narcotics and explosives, dogs are considered the gold standard, as they are efficient, cost effective, and can be more sensitive than some analytical methods (15, 17). However, the utility of canine odor detection has been scrutinized as variations in breed, physiology, training, handling, and study design have been noted to have a significant effect in accuracy and sensitivity (18–22). Odor detection dogs are typically trained over a prolonged period (typically at least 6 months of 3–5 days per week of 20–40 sessions per day for a dog with no prior scent work training) in a controlled laboratory setting to differentiate between target odor and control samples (12, 13, 17). These dogs are often trained using a reward-based system, non-restrictive search protocols, and multichoice systems (23). These training methods result in a high level of reported detection accuracy and specificity in a controlled setting, however canine odor detection accuracy and specificity may decrease when translated to open search environments (21). While the training of an odor detection dog to detect violative drug residues in food products to a high level of accuracy and sensitivity may involve a substantial time commitment, it offers the advantage of minimal equipment and trained personnel for sample analysis. Low frequency training, which this study defines as training of dogs 1 day per week for a limited number of sessions, may be a practical method for preparing on-farm working dogs for odor detection tasks in resource-constrained settings, where daily or intensive training may not be feasible. Dogs have demonstrated robust odor memory and, and previous studies have shown that dogs can retain target odor detection capabilities through the use of low-frequency reinforcement methods following initial training (24, 25). Additionally, in contrast to conventional odor detection training for working dogs, canines doing this type of work in a farm environment might benefit from a less controlled environment during the training process to be successful.

Extra-label drug use of antimicrobials is common in minor food producing species (such as sheep and goats) and has been identified as a risk factor for violative drug residues in milk of treated dairy cattle (26). Florfenicol, a broad spectrum amphenicol antibiotic, is commonly used in an extra-label manner for medical treatment of goats in the US where there is zero tolerance for any florfenicol residues (or its marker residue florfenicol amine (FA)) in milk (27). Currently there are no Food and Drug Administration (FDA) approved rapid assay tests for detecting florfenicol or FA residues in goat milk (28). However, using ultra-performance liquid chromatography with tandem mass spectrometry (UPLC-MS/MS) [limit of detection (LOD) = 3 ppb], florfenicol and FA residues have been found to be detectable in goat milk following extra label drug use for up to 30 days following the last dose and up to 33 days using a commercial rapid assay test validated for detection of florfenicol cow milk samples (LOD = 1 ppb) (27). Due to the prolonged period that florfenicol residues can remain detectable in milk samples, repeated testing of samples to ensure complete depletion would incur significant cost and labor. Therefore, it would be economically advantageous for producers and/or small farm operations to have access to a rapid, inexpensive method of screening milk for florfenicol residues to determine if further testing via validated methods is indicated. The objective of this pilot study was to evaluate if companion dogs of various breeds with limited experience could detect florfenicol residues in goat milk using a relatively low investment (compared to conventional odor detection training methods) training protocol.

## 2 Materials and methods

### 2.1 Animals

Eight dogs (Table 1) were selected for this pilot study from a group of privately owned companion dogs that were enrolled in a weekly canine scent detection course led by a Certified Canine BioDetection Dog Trainer (CBDT) with over 20 years of experience training sport dogs. The CBDT selected the 8 healthy dogs given their prior performance imprinting on odors. The dogs were trained using positive reinforcement and their owners demonstrated handling techniques that were conducive to successful training outcomes. The dogs remained healthy throughout the study period. When not participating in either imprinting or test sessions for this protocol, the dogs were maintained by their owners in their regular home environment, including their normal exercise routine. Additional odor detection training with florfenicol/FA residues samples outside of the experimental study was not allowed in order to standardize the dog’s training experiences with florfenicol/FA samples.

**Table 1:** Study ID number, breed, age, sex, reproductive status, and experience level at scent detection (as determined by a professional dog trainer) for companion dogs (*n =* 8) enrolled in a drug residue screening study.

Study ID	Breed	Age	Sex	Experience level
1	Australian Cattle Dog	2	Female Spayed	Experienced
2	Belgian Malinois	5	Male Intact	Intermediate
3	Labrador Retriever	9	Female Spayed	Experienced
4	Labrador Retriever × Standard Poodle	3	Male Castrated	Experienced
5	Dutch Shepherd	3	Female Spayed	Experienced
6	Nova Scotia Duck Tolling Retriever	6	Male Intact	Experienced
7	Rottweiler	3	Female Spayed	Experienced
8	Labrador Retriever × Miniature Poodle	2	Male Castrated	Novice

### 2.2 Training room and apparatus

**Figure 1:**
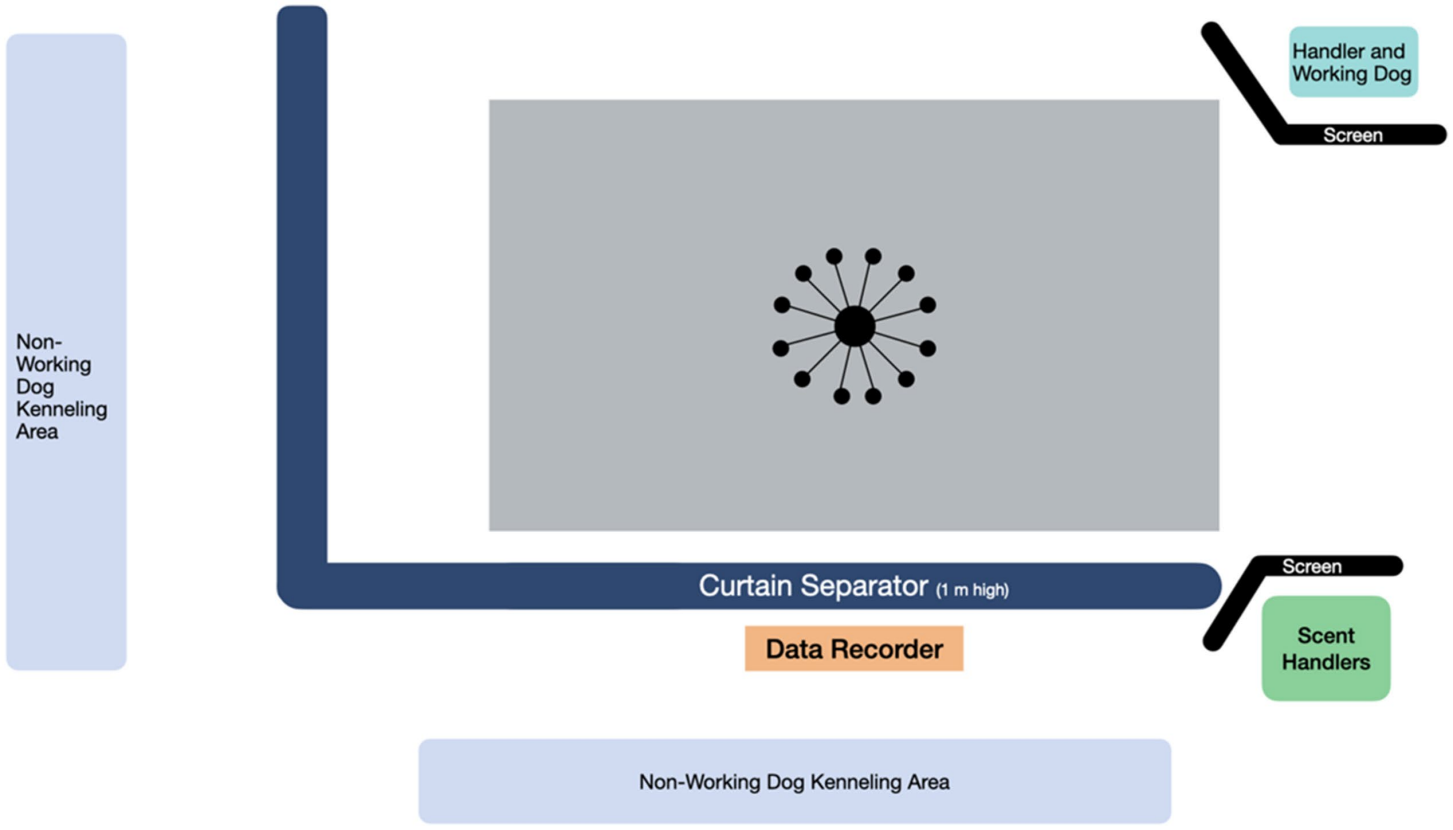
Space diagram for the area used during the imprinting study phase and sample testing study phases 1 and 2.

**Figure 2:**
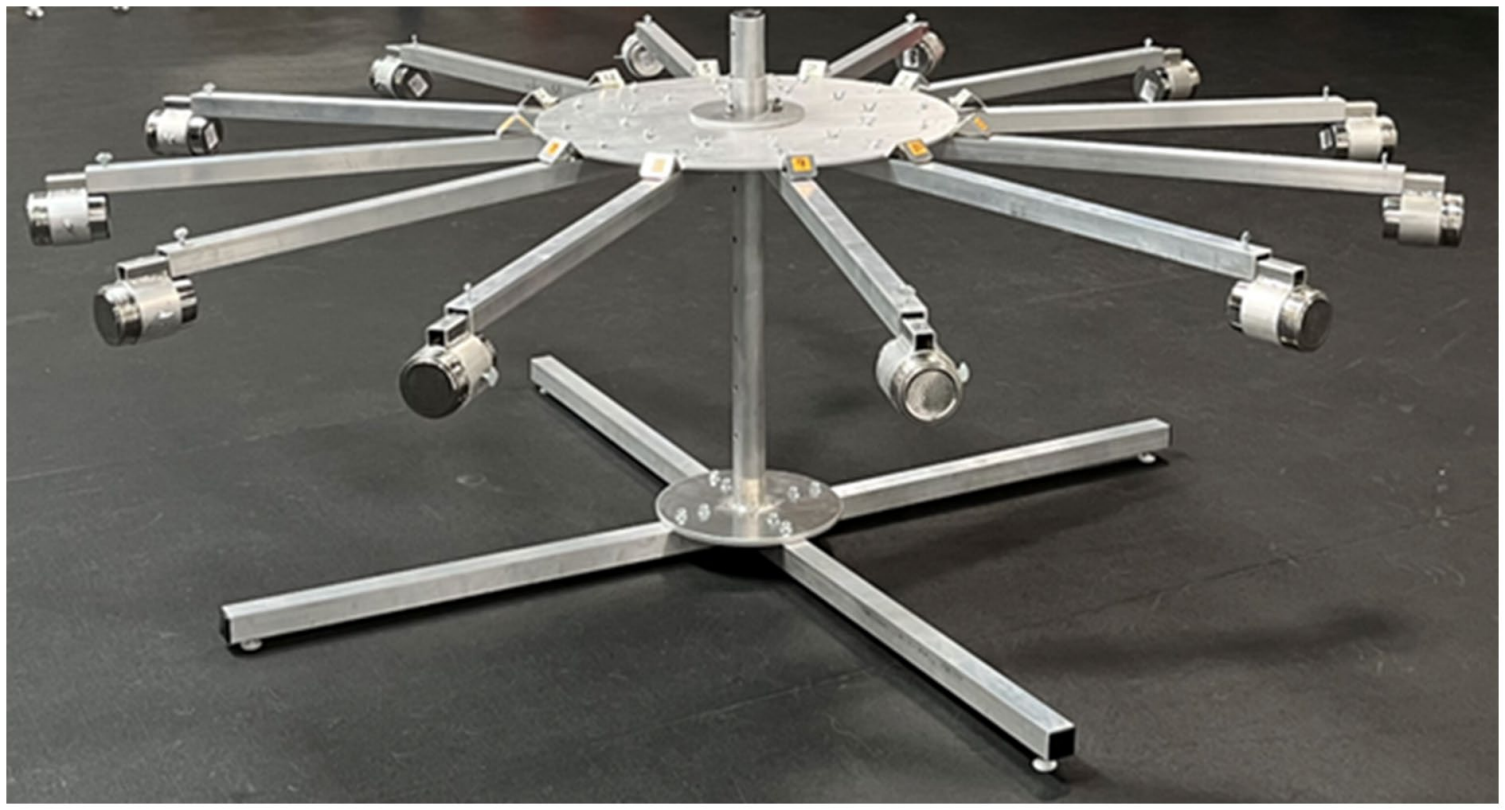
Sample carousel apparatus used in both the imprinting study phase and sample testing study phases 1 and 2.

Imprinting and testing was conducted in an air-conditioned mixed use indoor space where the dogs were currently undergoing training for their sport odor detection course. The space contained a specific fenced off area dedicated to odor detection training activities (9.5 m × 12 m) (Figure 1). The room temperature ranged from 18.9–22.8°C, and relative humidity ranged from 45 to 65% on the days when the dogs were participating in the study. The space had rubberized floor mats, which were swept and cleaned daily. The sample apparatus used for both imprinting and testing was the Detection Training Carousel (TDK9 Detection Training Carousel, Smithsburg, MD). The sample carousel apparatus (Figure 2) consisted of 12 removable arms with removable 10 oz. stainless steel scent cans with stainless steel mesh lids supplied by the same manufacturer (TDK9 Detection Training Carousel, Smithsburg, MD).

The sample carousel apparatus was cleaned using 75% alcohol sanitizing wipes (MagiCare Hand Sanitizer Wipes-Disposable 75% Alcohol Wipes, Manufacturer: Chaozhou Cecilia Technology Co. Ltd) and allowed to air dry prior to study initiation and prior to each study day. The cans were washed prior to each study day using the following multi-step procedure. The cans were soaked for 2 h in a commercial laboratory and surgical instrument cleaner containing 2-(2-butoxyethoxy) ethanol and dioctyl sulfosuccinate sodium salt (7X Cleaning Solution, manufacturer: MP Biomedicals, LLC) per labeled directions. The cans were then scrubbed and rinsed three times with hot water and three times with de-ionized water using a new sponge to avoid cross-contamination. Cans were subsequently dipped in 75% ethyl alcohol and allowed to air dry prior to transport to the study site in a clean lidded plastic tote used only for this study purpose.

### 2.3 Sample preparation, sample handling, and study protocol

Florfenicol amine reference standards used for the imprinting samples were obtained from a commercial chemical supplier (Cayman Chemical, Ann Arbor, MI). A stock solution for FA, was initially made at a concentration of 1,000 μg/mL by dissolving the dry crystalline solids in methanol. The FA stock solution was then serially diluted with methanol to form three additional stock solutions at concentrations of 100, 10, and 1 μg/mL. The FA stock solutions were then diluted either 1:100 or 1:200 in 100% de-ionized water to form five spiked solutions at concentrations of 10, 50, 100, 1,000, and 5,000 ng/mL. The spiked solutions were made fresh daily. Imprinting samples were prepared by pipetting 50 μL of spiked liquid solution onto a dry cotton tip swab and then allowed to dry isolated from other samples (with 1 meter between samples) for a minimum of 12 h prior to use. Prepared dried samples were place in a 2 mL glass vial that was sealed with a screw top lid during transport to the study site. Prepared dry samples were disposed after 24 h and a new batch was made for each imprinting session.

Clean nitrile gloves were always worn when handling the scent cans or when in contact with the sample carousel apparatus. To prevent a through draft or cross venting of samples, the gap opening at the back of each of the 12 arms on the sample carousel was blocked. Old gloves were removed and new gloves applied after touching any surface or anything that was not the sample carousel apparatus or a clean, unused scent can. To prevent cross-contamination, scent cans that contained the target odor (FA or incurred milk sample) were only moved by a single individual on each study day. Imprinting or study samples were added to empty clean cans at a minimum of 15 min prior to imprinting or testing to allow the sample odor to equilibrate.

A randomizer program (The Random Number Generator; Developer Nicholas Dean, Apple App Store Downloaded 4/17/2023) was used to determine the run order of sample and distractor locations in the wheel and dog run order for both imprinting/training and testing. Four individuals were engaged during the imprinting and testing of the dogs. One person (the CBDT, “dog handler”) removed the dog from the kennel, cued the dog to search during trials, interpreted if the dog alerted on a can, verbally called out the can number if a dog alerted on a can, and provided positive reinforcement for correct responses. The second person (“target odor handler”) was responsible for moving the scent can that contained the target odor between trials. The third person (“blank sample handler”) was responsible for moving the scent cans that did not contain the target odor between trials. The fourth person (“data recorder”) recorded the dog’s response and indicated to the dog handler whether to reward or not reward the dog for its alert during test runs.

The data recorder recorded verbal response from the handler of the canine’s response as a yes or no alert, and it was also indicated if the dog did not sniff the target odor (DNS). Dog sniffs of the distractors were recorded as sniffed, did not sniff (DNS) and distracted sniff (DS). If the dog did not sniff the distractor, it was not marked on the record sheet. To maintain double blinding, the data recorder noted all dog responses prior to any verbal comment or physical action by any of the other individuals in the room. The two individuals moving the scent cans turned their back on the sample carousel apparatus and handler during testing to not subconsciously give cues to identify the contaminated sample during testing. The dogs were leashed for the initial imprinting runs, but as the dogs became more accustomed to searching the training apparatus, the leash was removed, and the dog was allowed to search independently with the handler standing at a short distance away.

### 2.4 Dog *in vitro* sample imprinting

All dogs were trained using positive reinforcement, and were rewarded with a high value treat and the word “yes” at each correct on-target nose-hold. In the initial imprinting stage, the dogs were presented with three scent cans (one containing a FA swab and a dog treat, and two cans without any samples), and were trained to perform a nose-hold alert in front of the scent can containing FA. The unblinded imprinting study phase consisted of FA swabs presented in descending order of concentration 5,000–10 ppb with 20 runs per sample concentration level (Table 2). For each odor level, the dogs were run 5 times on the sample carousel apparatus and then returned to their crate for rest and more cans were added to the apparatus. Once all arms filled with cans, odor distractors were added. Odor distractors, items in the environment or sample preparation that may distract the dog while working, were introduced to teach the dog to focus on the target odor regardless of external stimuli. Distractors used were control untreated goat milk, 75% alcohol sanitizing wipe, methanol, pipette tip, glove, cotton tip, sharpie on paper, and unscented laboratory cleaner (7X Cleaning Solution). Low frequency imprinting training sessions were held once weekly with 5–10 sessions per day for nine consecutive weeks to correspond with the dogs’ normal training class schedule and owner commitments. Since the imprinting/testing sessions were only held once weekly, the dogs were allowed one unblinded warm-up run with a sample of a known high concentration prior to each testing session to ensure the dog was willing to work.

**Table 2:** *In vitro* training concentrations used to imprint dogs on florfenicol amine (FA) samples using olfaction.

Training level	Composition	Accuracy Mean (range)
1	FA (5,000 ppb) vs. 2 empty scent cans	0.79 (0.67–0.95)
2	FA (5,000 ppb) vs. 1 distractor scent can (methanol)	0.83 (0.59–1)
3	FA (1,000 ppb) vs. 2 distractor scent cans (glove, pipette tip)	0.75 (0.53–1)
4	FA (500 ppb) vs. 2 distractor scent cans (glove, pipette tip)	0.85 (0.62–0.96)
5	FA (100 ppb) vs. 2 distractor scent cans (methanol, blank milk)	0.87 (0.64–1)
6	FA (50 ppb) vs. 2 distractor scent cans (pipette tip, felt tip marker)	1 (1)
7	FA (10 ppb) vs. 2 distractor scent cans (methanol, blank milk)	1 (1)

Once each dog had imprinted on the respective concentration level 20 times, the handler was blinded to the target odor sample location and the dogs were tested on samples. For the test runs, the dog was presented with 2 odor distractors, 9 empty scent cans, and 1 positive target odor sample. The order of the scent cans was determined by a random number generator for each run. The dogs were allowed to sniff all cans twice, and if dogs were unable to decide/alert on a can they were returned to their crate, and the sample was reset. Dogs were tested 3 times on each sample. The dog was only rewarded once the location was announced by the handler and confirmed correct by the recorder, and the dog was only allowed to move onto the next florfenicol detection level if they correctly identified the target odor sample location on the test run. If the dog performed a nose-hold on an incorrect sample, the dog was ignored by the handler and no negative reinforcement was used. If the dog was unable to identify the correct sample they were imprinted again (minimum 5 imprint runs) and then retested on the target level concentration.

### 2.5 Dog *in vivo* sample imprinting

Once the dogs had successfully imprinted on the FA analytical standard solution for concentrations between 5,000–10 ppb, the imprinting process was repeated for florfenicol and FA in goat milk using samples of a known concentration from a previous study (27). Samples had been stored between 877 and 897 days post- collection at −70°C and were thawed and aliquoted into 5 mL samples. Samples were kept at −70°C between trial dates and thawed immediately prior to use, and the samples had never been thawed prior to use for this study. The imprinting process was repeated because the dogs were initially not able to detect residue positive incurred goat milk samples with combined florfenicol and FA. Therefore, the imprinting process was repeated for the in-vivo contaminated milk samples in the same fashion as the *in vitro* samples. The dogs were imprinted on 11 contaminated milk samples of a known concentration in descending order (combined florfenicol and FA concentrations range from 1443.30 ppb to 17.44 ppb). Since this was a pilot study, the 11 samples were chosen based on available samples from a prior goat milk pharmacokinetic study that had concentration levels the prepared range of spiked imprint samples with a minimum of two available samples per concentration category.

### 2.6 Olfactory detection testing

Olfactory detection testing of incurred florfenicol and FA residue goat milk samples was conducted in the same area as the imprinting sessions using the same sample carousel apparatus. In study phase 1, each dog was tested on the same 11 florfenicol contaminated goat milk samples of a known concentration, where each set consisted of 1 positive sample, 2 randomly assigned odor distractor samples, and 9 empty scent cans (Table 3). Three runs were performed per dog for each round/sample concentration, with the position of the positive sample and distractor cans randomized for each run within each round. Like the imprinting stage, the dog was only rewarded once the location was announced by the handler and confirmed correct by the recorder to minimize diminishment of the search behavior.

**Table 3:** Order of sample presentation, combined concentration of incurred florfenicol and florfenicol amine (FA) residues in goat milk samples, and distractors used for each dog (*n =* 8) for detection of incurred florfenicol residues in goat milk samples in study phase 1.

Sample order	Combined florfenicol and FA milk concentration (ppb)	Combined florfenicol and FA concentration category	Distractor 1	Distractor 2
1	162.51	3	Pipette tip	Cotton tip applicator
2	551.25	4	Plastic bag	Laboratory cleaner
3	66.40	2	Plastic bag	Blank milk
4	173.55	3	Blank milk	Felt tip marker
5	963.07	5	Pipette tip	Blank milk
6	46.13	2	Nitrile glove	Blank milk
7	501.29	4	Felt tip marker	Sanitizer wipe
8	17.96	1	Blank milk	Nitrile glove
9	17.44	1	Laboratory cleaner	Blank milk
10	23.77	1	Blank milk	Pipette tip
11	1443.30	5	Cotton tip applicator	Sanitizer wipe

Between each run, the handler and dog were placed behind a curtain approximately 2.6 m away while the positive sample and distractor scent cans were moved to the new randomized locations. Following each round, the dog was placed in its crate, and the scent cans were then reset. The next dog in the randomized order was collected to begin its round using the same florfenicol incurred residue goat milk sample. All dogs were presented with the same distractors in the same randomized scent can placement in each run. Scent cans that were licked or were noted to have saliva/nasal secretion were replaced with clean scent cans (including lids) following each round. Testing for study phase 1 lasted for 2 days (with each day 1 week apart). A total of 3 runs/dog for samples 1–4 were performed on day 1, and 3 runs/dog for samples 5–11 were performed on day 2. The randomized order of dogs was the same for both testing days.

Based on the dogs’ perceived performance in study 1 as evaluated by the CBDT trainer, 3 dogs (Dog IDs 2, 3, and 8) were selected to participate in study phase 2 of the detection test. In study phase 2, the dogs were presented with 11 additional florfenicol/FA contaminated goat milk samples that had been previously evaluated and found to be positive by a rapid residue detection test (RRDT; Charm® FLT; Charm Sciences Inc., Lawrence, MA) (27). These samples had very low combined florfenicol and FA concentrations (combined concentrations ranging from <2–14.46 ppb) as evaluated by UPLCMS/MS and were near the detection limit (2 ppb) for the rapid residue detection test (Table 4). Like study phase 1, each set consisted of 1 positive sample, 2 randomly assigned distractor samples, and 9 empty scent cans in study phase 2. Three runs were performed per dog for each round/sample concentration over a single day, with the position of the positive sample and distractor cans randomized for each run within each round.

**Table 4:** Order of sample presentation, concentration of combined florfenicol and florfenicol amine (FA) incurred residues in goat milk samples, and distractors used for each dog (*n =* 3) for detection of florfenicol incurred residues in rapid residue detection test (Charm^®^ FLT, Charm Sciences Inc., Lawrence, MA) positive goat milk samples in study phase 2.

Sample order	Combined florfenicol and FA milk concentration (ppb)	Distractor 1	Distractor 2
1	9.36	Blank milk	Cotton tip applicator
2	3.29	Blank milk	Nitrile glove
3	14.46	Blank milk	Plastic bag
4	6.59	Blank milk	Felt tip marker
5	< 2	Blank milk	Laboratory cleaner
6	2.0	Blank milk	Pipette tip
7	8.03	Blank milk	Sanitizer wipe
8	6.52	Blank milk	Nitrile glove
9	6.24	Blank milk	Felt tip marker
10	3.4	Blank milk	Felt tip marker
11	4.76	Blank milk	Sanitizer wipe

### 2.7 Data analysis

The olfactory detection performance of each dog was assessed as follows: (1) true positive: the dog indicates the target odor in the manner in which it was trained (“nose hold” response), (2) true negative: the dog does not alert in the absence of the target odor, (3) false positive: the dog alerts to a non-target position (empty can/distractor), (4) false negative: the dog did not alert in the presence of the target odor. The dogs’ accuracy was calculated based on the number of correct assessments (true positive + true negative) over the number of all assessments (true positive + true negative + false positive + false negative) of the test data. The dogs’ true-positive rate was calculated based on the number of correct assessments (true positive) over the number of alerts (true positive + false positive).

### 2.8 Statistical analysis

As this was a pilot study, limited statistical analysis was performed. Accuracy, sensitivity, specificity, and their associated exact 95% binomial confidence intervals were calculated using GraphPad Prism 10.3.1 (GraphPad Software, Boston, MA) to evaluate detection accuracy, sensitivity, and specificity based on combined florfenicol/FA concentration for study phase 1.

Concentrations of combined incurred florfenicol and FA residues in goat milk samples were grouped into 5 categories for study phase 1: Category 1 (<40 ppb), Category 2 (41–100 ppb), Category 3 (101–500 ppb), Category 4 (>501–900 ppb), and Category 5 (>901 ppb) (Table 3). Mixed-effects logistic regression was performed in Stata BE 17.0 (StataCorp LLC, College Station, Texas, USA), with dog and run as random effects, to determine the odds of success in accurately detecting florfenicol residues in contaminated goat milk between concentration categories; results are presented as odds ratios (OR) and 95% confidence intervals (CI). An unpaired *t*-test was performed in GraphPad Prism 10.3.1 to evaluate differences in the average time to nose-hold between outcomes (true positive vs. false positive) for study phase 2. All results were considered significant at *p* < 0.05.

## 3 Results

Each dog (*n =* 8) completed 33 test trials consisting of 3 rounds for each of the 11 florfenicol incurred residue goat milk samples plus 2 distractors for study phase 1. Overall detection accuracy for all dogs across all concentrations and all runs in study phase 1 was 0.80 (95% confidence interval (CI) 0.74–0.86), overall sensitivity was 0.70 (95% CI 0.65–0.76), and overall specificity was 0.86 (95% CI 0.82–0.88), with individual dog data presented in Table 5. Of the total 76 false positive alerts in study phase 1, 58/76 false positive alerts were on blank goat milk, 14/76 false positive alerts were on empty canisters, and 4/76 were false positive alerts on sanitizer wipes. Overall detection accuracy, sensitivity, and specificity for combined florfenicol and FA incurred residues in goat milk samples for study phase 1 based on concentration are noted in Table 6.

**Table 5:** Detection accuracy, sensitivity, and specificity for combined florfenicol and florfenicol amine incurred residues in goat milk samples based on dog ID for *n =* 8 dogs in study phase 1.

Dog ID	Overall accuracy	Overall sensitivity	Overall specificity
1	0.74	0.61	0.80
2	0.92	0.88	0.94
3	0.74	0.61	0.80
4	0.88	0.82	0.91
5	0.78	0.67	0.83
6	0.78	0.67	0.83
7	0.74	0.61	0.83
8	0.86	0.79	0.89

**Table 6:** Detection accuracy, sensitivity, and specificity for florfenicol incurred residues in goat milk samples based on combined florfenicol and florfenicol amine (FA) concentration for *n =* 8 dogs in study phase 1.

Combined florfenicol and FA concentration (ppb)	Combined florfenicol and FA concentration category	Accuracy mean (95% CI)	Sensitivity mean (95% CI)	Specificity mean (95% CI)
17.44	1	0.70 (0.47–0.92)	0.54 (0.21–0.87)	0.77 (0.61–0.94)
17.96	1	0.59 (0.40–0.77)	0.37 (0.10–0.65)	0.69 (0.55–0.83)
23.77	1	0.81 (0.65–0.96)	0.71 (0.48–0.94)	0.85 (0.74–0.97)
46.13	2	0.72 (0.53–0.92)	0.58 (0.29–0.87)	0.79 (0.65–0.94)
66.40	2	0.72 (0.50–0.94)	0.58 (0.26–0.91)	0.79 (0.63–0.95)
162.51	3	0.89 (0.79–0.99)	0.83 (0.68–0.98)	0.92 (0.84–0.99)
173.55	3	0.75 (0.57–0.94)	0.63 (0.35–0.90)	0.81 (0.67–0.95)
501.29	4	0.86 (0.64–1.0)	0.79 (0.46–1.0)	0.90 (0.73–1.0)
551.25	4	0.95 (0.81–1.0)	0.92 (0.72–1.0)	0.96 (0.86–1.0)
963.07	5	0.92 (0.78–1.0)	0.88 (0.67–1.0)	0.94 (0.83–1.0)
1443.30	5	0.97 (0.91–1.0)	0.96 (0.86–1.0)	0.98 (0.93–1.0)

Results of the mixed-effects logistic regression analysis revealed that the odds of success in detection of category 5 (>901 ppb) combined florfenicol and FA concentrations was significantly higher than that of category 1 (<40 ppb) concentrations (OR 9.8, 95% CI 3.2–30.7, *p* < 0.001). The odds of success at detecting a category 4 concentration (501–900 ppb) were also significantly higher than detecting a category 1 concentration (OR 5.2, 95% CI 2.0–13.3, *p* = 0.001). There was no significant difference in the odds of success for detecting a category 3 concentration (101–500 ppb, OR 2.1, 95% CI 0.96–4.6, *p* = 0.07), or category 2 concentration (41–100 ppb, OR 1.2, 95% CI 0.56–2.5, *p* = 0.65) when compared to category 1 concentrations.

Each dog (*n =* 3) completed 33 test trials consisting of 3 rounds for each of the 11 low concentration, RRDT positive florfenicol/FA contaminated goat milk samples plus 2 distractors for study phase 2. There was no significant difference in the time to nose hold between true positive and false positive outcomes for *n =* 3 dogs in study phase 2 (*p* = 0.43). Of the 35 false positive alerts in study phase 2, 34/35 were to blank goat milk, and 1/35 was to an empty canister. Mean detection accuracy for all dogs across all concentrations and all runs for RRDT positive samples in study phase 2 was 0.88 (95% CI 0.85–0.91), mean sensitivity was 0.82 (95% CI 0.73–0.88), and mean specificity was 0.91 (95% CI 0.86–0.94). For both Dog ID 2 and Dog ID 8 in study phase 2, accuracy was 0.90, sensitivity was 0.85, and specificity was 0.92. For Dog ID 3 in study phase 2, accuracy was 0.84, sensitivity was 0.76, and specificity was 0.88.

## 4 Discussion

In this pilot study, it was demonstrated that companion dogs with low-frequency training for canine olfactory odor detection were able to detect incurred residues of florfenicol and its metabolite FA, in goat milk samples with a mean sensitivity of 0.70 (95% CI 0.65–0.76) and a specificity of 0.86 (95% CI 0.82–0.88). Furthermore, the potential utility of companion dogs undergoing low-frequency training could be a cost-effective screening method for detecting florfenicol residues in goat milk samples since the specificity was >0.80 for all combined concentrations of florfenicol and FA. However, benefits of using dogs for florfenicol residue detection in goat milk samples diminished at low concentrations (average 0.38 at 17.96 ppb) compared to high concentrations (0.96 at 1443.30 ppb), and detection of residues at low concentrations was subject to substantial interindividual variability. Given the success of using professionally trained working dogs for odor detection, this issue might be mitigated with a more robust training model but would need to be investigated further.

In our pilot study, there are multiple factors that may have influenced the variability of dogs being able to detect florfenicol/FA residues. When detecting incurred florfenicol/FA residues in goat milk, the odds of success only significantly increased for the two highest concentration groups compared to the odds of success for the lowest concentration group. Considering that the most common false positive alert in this study for both study phases 1 and 2 was the blank goat milk matrix, there was potentially some failure in training/imprinting phase, where dogs generalized to the odor of goat milk rather than to the odor of florfenicol/FA if there was not a positive sample in the sample carousel apparatus and the dogs were seeking to be rewarded with a treat. Generalization and discrimination can impact the dog’s ability to regulate how they respond to the target odor and consequently the non-target odors (29, 30). This could be improved by including more blank milk samples in the carousel during the imprinting training phase to thoroughly proof the dogs off the blank milk samples. Additionally, matrix odor interference may have been the cause of false positives on blank goat milk. Matrix odor interference of the goat milk could potentially have overwhelmed the senses of the dogs olfactory system or masked the odor of florfenicol target odor and led to increased false positives (30). Another strategy to reduce false alerts may be accomplished by including a higher frequency of blank trials, where only distractors odors are available, to encourage operational performance (31). Blank goat milk was used as a distractor in 3/3 category 1 concentration samples, 2/2 category 2 concentrations, 1/2 category 3 concentrations, 0/2 category 4 and category 5 concentrations. It might also be presumed that the increase in odds of success for category 4/5 concentrations in comparison to category 1 may have been due to decreased challenge of the detection task at higher concentrations because of the lower prevalence of a blank milk distractor in those sample sets and there wasn’t as high of a likelihood of the dogs cueing in the next most likely odor (goats milk) that earned them a reward since blank goat milk samples were not distractors for those categories. For future studies, blank goat milk samples should always be included as a distractor for all concentrations.

The need for additional imprinting when moving from the FA spiked to the incurred florfenicol and FA goat milk samples may have been due to interference of florfenicol parent drug. As the florfenicol parent drug was not present in the spiked samples used for the initial imprinting stage, it is most likely the cause of the need for additional imprinting when moving to the incurred residue samples. Florfenicol is metabolized to FA through two bioconversion pathways, the first through monochloroflorfenicol, and the second via florfenicol alcohol with or without generation of the florfenicol oxamic acid intermediate (32). Due to the changes in chemical structure between florfenicol and FA, it is likely that the additional imprinting required for this study is due to the combined presence of florfenicol and FA in the incurred residue samples. Another theory could be that the dogs did not have enough experience to generalize the spiked samples and transfer detection cuing to the incurred samples. However, that seems less likely of an explanation since three dogs with the lowest overall sensitivity for florfenicol/FA detection in goat milk samples in this study were considered advanced level, while three dogs with the highest sensitivity for florfenicol/FA were advanced, intermediate, and novice. The dogs finding difficulty in generalization of odors from training samples to test samples in this study is not considered unusual, since previous studies have also noted inter-individual variability in the ability to generalize from training samples to test samples (12, 33). The challenge in transitioning from spiked to incurred samples in this study may have been exacerbated by the once weekly training model. However, canine olfactory memory has been documented to be quite robust, with odor discrimination to learned odors lasting for at least 12 months (25, 34). Furthermore, it has been shown that dogs can successfully maintain olfactory memory and target indication behavior under intermittent reinforcement schedules, indicating that low-frequency reinforcement models could possibly be effective once an odor is learned (35). However, since the dogs in our study were not trained on a more frequent and intense basis, such as what is done with professional odor detection dogs, perhaps there was decreased target indication behavior without continued reinforcement until the odor detection was robustly established. Another possibility is that there was a slight difference in the chemical structure/odor between spiked samples versus incurred samples. Interestingly, there was a subjective interpretation that the imprinting of dogs on incurred samples was much quicker compared to the spiked samples, perhaps indicating that the dogs were already familiar with the FA odor but needed to be trained to cue on the combined florfenicol and FA odor present in the incurred residue goat milk samples. Finally, since there are a number of behavior, environmental, and physiologic factors that contribute to canine olfactory memory (36), the inter-individual performance variation in this study could have been due to a complex of variety of factors.

For our pilot study, since there were limited numbers of incurred residue goat milk samples, a few of the incurred goat milk samples used in the secondary imprinting stage were also used in the blind testing phase (study phase 1). Given the repetition of a limited number of samples for both imprinting and blind testing, it was anticipated that the accuracy, sensitivity, and specificity reported in study phase 1 might be higher than if all samples in study phase 1 were novel samples. However, the 3 dogs that participated in study phase 2 with novel low-concentration samples had a comparable overall mean accuracy (0.88), sensitivity (0.82), and specificity (0.91) to their performance in study phase 1 [accuracy (0.84), sensitivity (0.76), and specificity (0.88)]. These results suggest that, at least for the subpopulation of selected high performing dogs, previous sample exposure was less important than reinforcement schedule for overall detection performance, and that increasing reinforcement led to improvement in detection accuracy, sensitivity, and specificity. Therefore, the reduced reinforcement schedule of this once weekly training model in pet dogs is more likely to have played a role in the lower sensitivity for florfenicol/FA detection than dog skill level, and increased reinforcement frequency during the initial imprinting stages may lead to higher sensitivity.

In study phase 2, when comparing the sensitivity of residue detection by the RRDT versus dog olfaction residue detection, the sensitivity of the three selected dogs was lower (0.82) than the manufacturer reported sensitivity of the RRDT (0.95) for samples in a cow milk matrix (37). Since the RRDT used to previously evaluate goat milk samples, that were also used for canine olfaction detection in study phase 2, has not been validated for a goat milk matrix, only samples that were positive on RRDT and had concentration verification by UPLC-MS/MS were used for our canine olfaction detection study (27). Even though there were limited numbers of high performing dogs (*n =* 3) used for study phase 2, preliminary results show that low-frequency training companion dogs might be able to detect florfenicol/FA incurred residues in goat milk samples as low as 2 ppb, and these dogs were able to discriminate the florfenicol/FA contaminated goat milk sample from blank goat milk at very low concentrations. While this pilot study demonstrates potential for using companion dogs for olfactory residue detection, there were other limitations to consider in addition to those previously discussed. For our study, a controlled indoor environment was used, and external confounders were minimized, which is optimal from a study design perspective. However, this does not replicate on-farm conditions where canine olfactory detection might encounter additional environmental variables such as fluctuating temperatures, humidity, animal distractors, and extraneous odors. In particular, fluctuations in environmental temperature may have a negative effect on the sensitivity of the dog’s ability to detect florfenicol residues. High temperatures may influence the ability and willingness of the dog to search, reduction of nasal mucosal fluidity and consequently interference with the function of the olfactory epithelium can occur from dehydration and panting of the working dog (38, 39). Consequently, lower temperature may affect the samples ability to odorize the scent can and be harder for the dogs to detect (40). Studies have found olfactory performance of dogs may be affected when testing indoors and outdoors, with better success in controlled indoor settings (40). If dogs are to be trained and tested in outdoor environment, increased effort and time will need to be taken to proof dogs off distractors and teach the dogs to focus on identifying the target odors. Limitations of previous canine olfaction studies have included lack of double blinding and inadvertent cuing by the dog handlers (18, 41). However, our study minimized the limitation of having multiple scent can handlers, by using a single experienced handler (and not the dogs’ owners) that was blinded to scent can location during the testing phases. Although not an objective of this study, determining a definitive detection threshold could not be performed since the samples used for this study had been analyzed previously. Finally, the inclusion of laboratory-oriented distractors, such nitrile glove, pipette tips, etc. might not accurately represent the range of odors dogs might encounter in real-world screening scenario, so expansion of distractor testing should be considered for future studies. Dogs used for food safety detection work are generally trained to have higher sensitivity as they need the ability to detect when a contaminant is present in a food item. With higher sensitivity false positives are more likely to occur, as such additional testing should be implemented to offset this (42). Law enforcement dogs on the other hand are generally trained to have a higher specificity in order to not falsely identify individuals or objects (43).

Since this was a pilot study, further studies are necessary to determine whether canine olfactory residue detection using companion dogs could be a confirmatory testing method for detecting violative residues in goat milk and what level of training commitment would be necessary to result in statistically significant increases in performance. Since the sensitivity of canine olfactory residue detection for florfenicol/FA residues in goat milk at low concentrations in this pilot study (mean 0.56 for concentrations <100 ppb in study phase 1, and 0.82 for concentrations <20 ppb for study phase 2) was less than the manufacturer reported RRDT sensitivity (0.95) or the limit of detection for the gold standard UPLC-MS/MS (LOD = 3 ppb) increased frequency of training might be necessary but once the investment of time and effort is made, the payoff might be beneficial. This pilot study demonstrated that use of low-frequency trained companion dogs for detecting florfenicol/FA residues in goat milk samples is promising based on the higher specificity in both study phases 1 and 2 and increasing sensitivity at higher florfenicol/FA concentrations. Companion dogs could be used for screening samples until concentrations fall into the lower concentration categories, where at that time use of the RRDT might be more appropriate for confirmatory testing if increased training does not yield better results. In resource-limited settings, where access to analytical instrumentation, trained personnel, and reagents may be constrained, the use of trained detection dogs could provide a cost-effective strategy by narrowing the number of samples requiring drug residue confirmation with formal laboratory analysis. However, the scalability of canine olfactory residue detection programs may depend on several practical factors, including training duration, handler expertise, and environmental conditions that can influence detection performance. These considerations are important when evaluating the feasibility of implementing canine detection systems in agricultural or food safety contexts.

In conclusion, this pilot study demonstrates that companion dogs, trained with low-frequency reinforcement, can detect incurred florfenicol and FA residues in goat milk with high specificity (0.86 on average). Advantages of using companion dogs for olfactory odor detection for drug residues in matrices intended for human consumption include streamlining monitoring efforts and minimizing the need for testing large numbers of samples, reducing reliance on costly laboratory equipment, and providing rapid, on-site screening methods for drug residues. Furthermore, by training companion dogs to perform initial screening for presence of drug residues in milk samples, developing nations might be able to enhance food safety and regulatory compliance, particularly in regions with limited access to farm side and/or advanced analytical equipment. Based on the outcomes reported in this study, strategies to train and improve the companion dog’s ability to discriminate between the blank and contaminated milk and prevent generalization and matrix odor interference should be explored further. The use of companion dogs to detect chemical residues may be an effective low-cost alternative for drug residue screening when compared to traditional highly sensitive detection methods such as spectroscopy, immunoassays, electrophoresis, and farm side rapid assay tests (44). The use of companion dogs to detect chemical residues may be an effective low-cost alternative for drug residue screening when compared to traditional highly sensitive detection methods such as spectroscopy, immunoassays, electrophoresis, and farm side rapid assay tests (44). While our pilot study did not definitively determine if canine olfactory detection using companion dogs could be a stand-alone confirmatory method, increasing the training commitment and refining imprinting protocols might improve detection performance and thus demonstrate higher promise for using companion dogs in food safety surveillance programs.

## Data Availability

The original contributions presented in the study are included in the article/supplementary material, further inquiries can be directed to the corresponding author.
